# Evolution of digital twins in precision health applications: a scoping review study

**DOI:** 10.21203/rs.3.rs-4612942/v1

**Published:** 2024-08-07

**Authors:** Yu Huang, Hao Dai, Jie Xu, Ruoqi Wei, Leyang Sun, Yi Guo, Jingchuan Guo, Jiang Bian

**Affiliations:** 1 Department of Health Outcomes and Biomedical Informatics, University of Florida, Gainesville, FL, USA; 2 Department of Pharmaceutical Outcomes and Policy, University of Florida, Gainesville, FL, USA

**Keywords:** Health Digital Twins, Real-world Data, Artificial Intelligence, Microsimulation, Agent-based Modeling

## Abstract

An increasing amount of research is incorporating the concept of Digital twin (DT) in biomedical and health care applications. This scoping review aims to summarize existing research and identify gaps in the development and use of DTs in the health care domain. The focus of this study lies on summarizing: the different types of DTs, the techniques employed in DT development, the DT applications in health care, and the data resources used for creating DTs. We identified fifty studies, which mainly focused on creating organ- (n=15) and patient-specific twins (n=30). The research predominantly centers on cardiology, endocrinology, orthopedics, and infectious diseases. Only a few studies used real-world datasets for developing their DTs. However, there remain unresolved questions and promising directions that require further exploration. This review provides valuable reference material and insights for researchers on DTs in health care and highlights gaps and unmet needs in this field.

## Introduction

Digital Twin (DT) is a novel approach in aiding decision-making to solve various real-world challenges and has attracted growing attention in both industry and research communities. The original use of the “twin” concept can be traced back to NASA’s Apollo mission in the 1960s, where engineers created a “living model” to simulate spacecraft. In 2010, John Vickers introduced the term “digital twin”, which consists of three components: a virtual system, a physical entity, and a bidirectional connection linking each other. While DTs have been widely studied in manufacturing, there is more and more interest in their applications in health care.

Recently, healthcare research has evolved from traditional reactive methods to proactive strategies [2–4]. Healthcare practitioners are trying to provide precision medicine, focusing on improving human health by evaluating individualized factors and acting on them [2]. The goal is to “target the right treatments to the right patients at the right time” [3]. Health digital twin, relying on artificial intelligence (AI) and machine learning (ML), is promising in this context. A health DT refers to the use of DT in health care, modeling patients, organs, pathophysiological systems, and/or other health-related entities (e.g., a hospital), to offer solutions in precision medicine, clinical trials, and public health.

DTs resemble other modeling techniques, such as microsimulation (MSM) and agent-based modeling (ABM). In health care, an MSM model simulates individual behaviors (“micro”, e.g., at the patient or household level) to estimate population-level effects^1,2^. MSM has been used in various disease analyses (e.g., dementia^3^ and oncology^4^). The National Cancer Institute (NCI)’s Cancer Intervention and Surveillance Modeling Network (CISNET) program has built MSM models to analyze cancer control interventions^5^. ABM, another simulation method, is focused on simulating the actions of targeted agents (e.g., patients) interacting with a specific environment. During the COVID-19 pandemic, ABM was used to model human activities and virus transmissions for assessing the impact of public health interventions (e.g., social distancing and vaccination strategies).^6–8^

While MSM and ABM have a long history in health care applications, discussions surrounding DTs have surged more recently along with the advancement of AI/ML and the proliferation of big data. Several existing efforts have provided up-to-date perspectives on DTs in health care^9–11^. A few review papers were published to discuss specific disease applications, such as multiple sclerosis^12^, cardiovascular disease^13^, COVID-19^14^, and the immune system^15^. Nevertheless, the existing work has only focused on discussing DTs, MSM, and ABM, separately, without drawing similarities and distinctions across the three. In addition, DTs studies have emerged in health care, however, there is a lack of clarification of the difference among DTs, MSMs, and ABM, and the summarization of the DTs in health. In this scoping review work, first, we aimed to bridge this gap by providing a comprehensive and nuanced picture of health DT literature, highlighting the differences and overlaps across these modeling techniques. In addition, we conducted a comprehensive review of individual studies in DTs for healthcare applications.

## Method

### Search strategy and selection criteria

In this study, we performed a two-phase literature review: the first phase focused on evaluating existing review articles related to DTs, MSM, and ABM; the second phase assessed individual studies about health DTs. For both phases, we adhered to the same methodology following the Preferred Reporting Items for Systematic Reviews and Meta-Analyses (PRISMA) guidelines, which included a comprehensive literature search, a review of abstracts and full texts, and data extraction from selected articles.

In the first phase, we aimed to examine the definition, scope, and progress of DTs by extracting review papers that discussed relevant techniques and concepts, including DTs, MSM, and ABM. We collected peer-reviewed publications from mainstream databases, including IEEE Xplore (n = 36), ACM Digital Library (n = 4), Web of Science (n = 57), PubMed (n = 158), and Embase (n = 25), using the search query: Title: (“survey” OR “review” OR “overview” OR “summary”) AND Abstract: (“digital twin*” OR “microsimulation” OR “agent-based simulation”) AND (“healthcare” OR “health” OR “infection” OR “disease*”) with filters limiting to studies published in the last decade (2013–2023). We then excluded papers that (1) were not review articles, (2) were not related to health care or medicine, or (3) were not written in English. A total of 25 review papers were included from 274 papers.

In the second phase, we focused on individual studies of DTs in health care, sourced from PubMed (n=611) and Embase (n=669). Our initial inclusion criteria specified studies that were (1) published within the past 10 years (2013–2023), (2) written in English, and (3) related to DTs in health care and biomedical applications (e.g., diagnosis, treatment, and drug discovery/development). We excluded studies that were (1) not peer-reviewed original research (e.g., pre-prints), (2) lacking full text, (3) commentaries, perspectives, or editorials, or (4) unrelated to the health care domain. After an initial screening of 940 papers, we discarded those that were not relevant (n=800) or did not meet our criteria (n=90), resulting in 50 papers selected for data extraction and final analysis.

**Figure 1.**
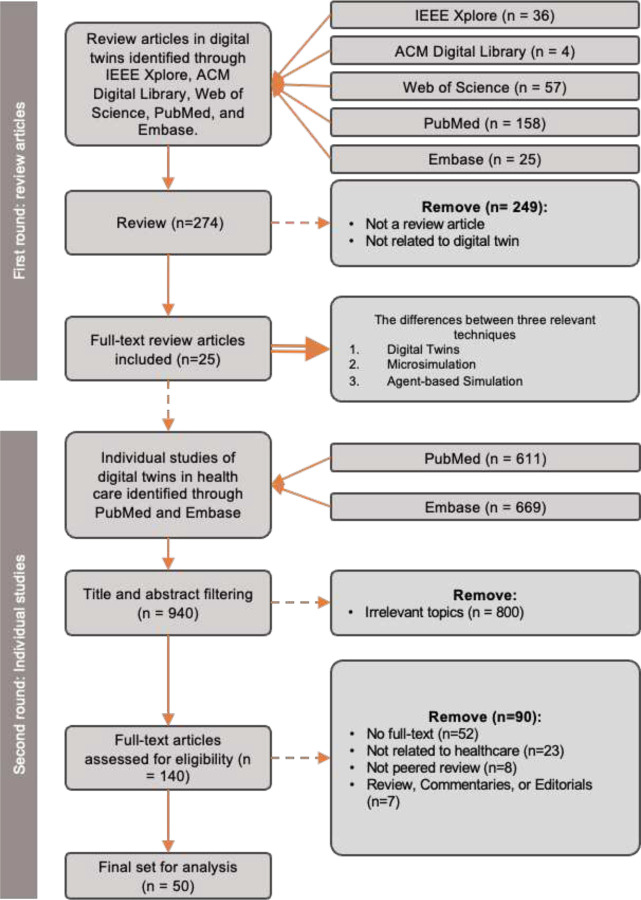
Flow diagram illustrating the process of study.

For both phases, all papers underwent a two-person independent review process for the inclusion and exclusion; and conflicts were resolved by a third reviewer. The details and outcomes of both the Phase 1 and Phase 2 reviews, following the PRISMA guidelines, are illustrated in Figure 1.

## Results

**Figure 2.**
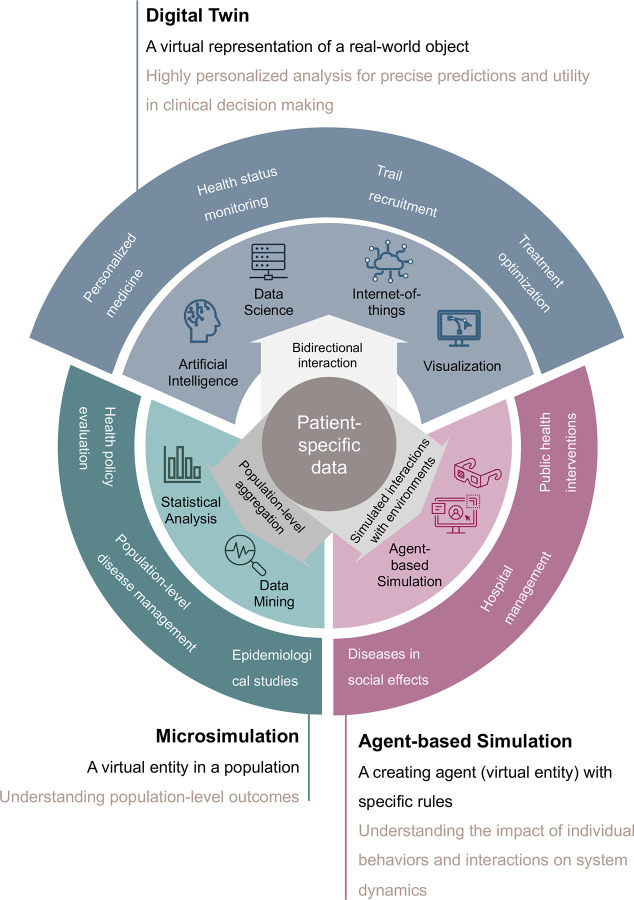
Differences among digital twins, microsimulation, and agent-based simulation in healthcare.

We summarized the characteristics (Figure 2) of DTs, MSM, and ABM, based on 25 review articles. These three modeling approaches have distinct ways of analyzing data and serve different purposes during applications. DTs rely on AI/ML and visualization techniques to create personalized models of real-world objects with data connections. MSM mainly leverages existing statistics and conducts analysis and draws evidence at the population level, while ABM focuses on the behaviors of agents in specific environments. It is worth noting that the analytical methods used in MSM and ABM can also contribute to the development of DTs.

In the Phase 2 review, we conducted a detailed analysis of the existing studies on health DTs, summarizing their types, foundational techniques, applications, and datasets:

### Types of DTs in Health care

**Figure 3.**
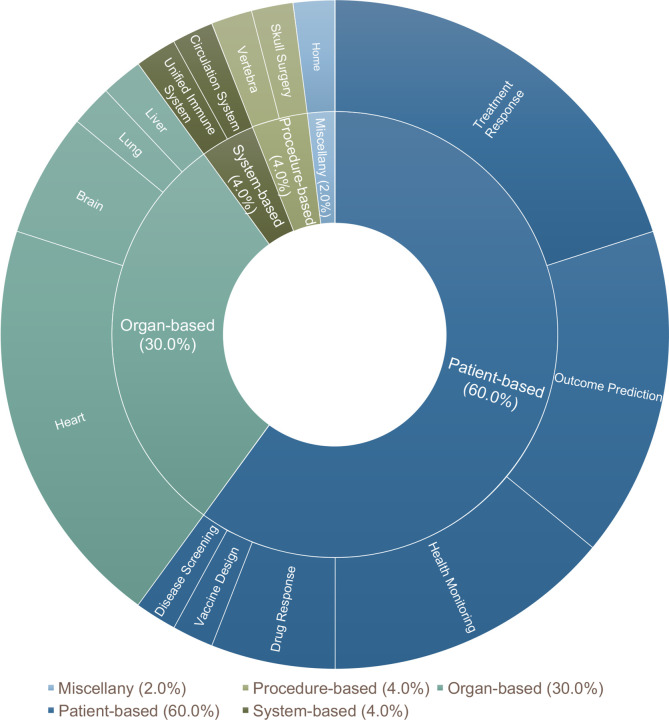
Types of current health digital twins.

As shown in Figure 3, current research on DTs in health care can be categorized into five classifications based on the physical entity they represent: organ-based (30%), physiological system-based (4%), patient-based (60%), procedure-based (4%), and miscellaneous (2%).

Health DTs for modeling specific organs were commonly used for improving health monitoring and treatment regimen optimization. In this classification, the majority of studies^16–25^ developed DTs of the heart (n=10), with fewer studies targeting the brain^26–28^ (n=3), liver^29^ (n=1), and lungs^30^ (n=1).

In DT application for physiological systems, Golse et al.^31^ created a DT to mimic the overall circulation system to estimate patients’ preoperative conditions and predict postoperative hemodynamic status. Similarly, Maleki et al.^32^ built a DT model of the immune system to inform clinical decisions.

Patient-level DTs replicate individual patients and are primarily focused on utilizing patient-DTs to support decision-making on therapeutics and interventions, including optimizing treatment strategies^33–38^, predicting chemotherapy responses^39^, and evaluating dietary interventions^40–43^. Another application focus is patient health and outcomes predictions using DTs, such as the onset of disease-specific brain atrophy^44^, the spread of COVID-19^45^, the risk of vertebral fracture^46^, the occurrence of metastases^47^, the progression of diabetic retinopathy and cataracts^48^, the auxiliary diagnosis of sepsis^49^, and long-term health management (e.g., life-course risk of multimorbidity)^50^. Additionally, there is a growing interest in creating patient DTs for health and vital sign monitoring, such as tracking and forecasting glucose^51–54^, blood velocity and pressures^55,56^, and body mass^57^. Being distinct from organ or physiological system-based DTs, patient-based DTs do not model an organ or physiological system directly. Nevertheless, patient-based DTs adopt a more comprehensive view of the human body and the surrounding environment, allowing for the simulation of physiological systems or organs as part of the patient-based DTs. A smaller segment of research was focused on building patient-based DTs for drug development, such as drug reactions^58,59^ and diffusion^60^. In addition, using data from actual human leukocyte antigen (HLA) haplotypes of ~22,000 individuals, Malone et al.^61^ employed a DT-type simulation to design vaccines for preventing COVID-19.

Two studies focused on procedure-based DTs, such as Ahmadian et al.^62^ simulated the cement injection process of the vertebra in a DT to predict vertebral compression fractures. Shu et al.^63^ proposed a DT framework, called Twin-S, to simulate skull surgery procedures. We also identified miscellaneous DTs beyond organs, physiological systems, patients, or procedures. Fahim et al.^64^ constructed a home-based DT to monitor the daily activities of the elderly.

### Core Models of Health Digital Twins

**Figure 4.**
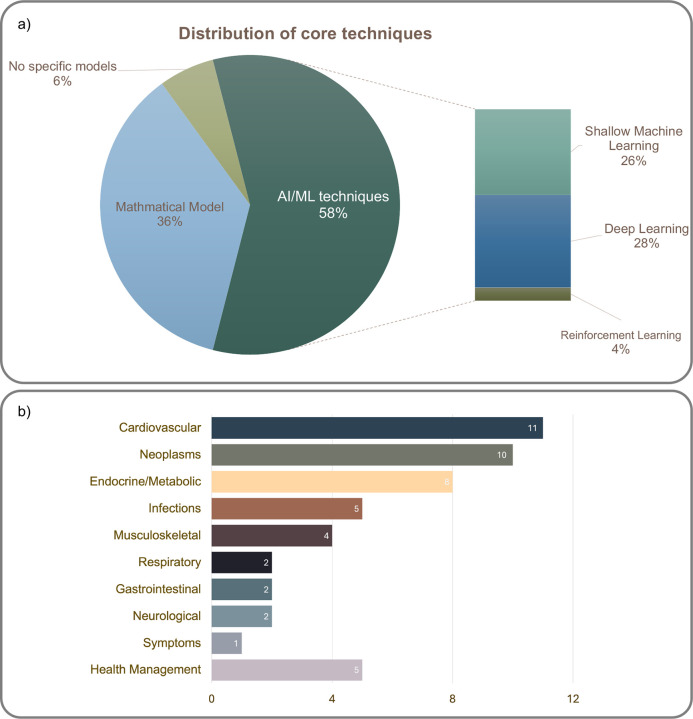
Techniques and applications of health digital twins. a) Core techniques to create health digital twins. b) Distributions of health digital twins in disease application domains.

The existing research employed both complex AI/ML techniques (58%) and simple statistical/mathematical models (36%) to build DTs (Figure 4-a). For instance, Lal et al. (2020)^33^ utilized Bayesian networks as the core of a DT to simulate treatment responses for sepsis patients. Malone et al. (2020) proposed a DT framework with variations of Support Vector Machines^61^ for COVID-19 vaccine design. Recurrent neural networks (RNN)^18,37,52,64^ and generative adversarial networks (GAN)^45,51,55^ were utilized for DT development in the analysis of complex data like sequential data (e.g., sensor readings and vital signs). When dealing with medical images, convolutional neural networks (CNN)^26,27,49^ and GAN^23,46,62^ are frequently used. Batch et al. (2022)^47^ integrated RNN and CNN to develop a cancer DT for metastasis detection based on information extracted from a sequence of patients’ past structured radiology reports. Additionally, reinforcement learning has been applied to optimize therapeutic regimens, including parameter adjustments for implantable cardioverter defibrillators^21^ and treatment strategies for cancers like oropharyngeal squamous cell carcinoma^35^.

Mathematical models often leverage prior knowledge (e.g., equations of cell model and tissue propagation) and/or existing statistics to create DTs. For example, Goodwina et al.^54^ proposed a metabolic DT framework based on a probabilistic model to optimize insulin dosing by simulating the blood glucose trajectories. Galappaththige et al.^22^ developed a cell model and tissue propagation equations to establish patient-specific cardiac electrophysiological models. Gillette et al.^16,17^ employed a reaction-eikonal model to create high-fidelity cardiac DTs that simulate ventricular electrophysiology. Wu et al.^39^ combined magnetic resonance imaging (MRI) data with biologically based mathematical models to generate patient-specific DTs for predicting and assessing treatment responses (e.g., chemotherapy). Qi et al.^38^ created virtual patients by incorporating realistic baseline tumor burdens, anatomical lesion distributions, non-target progression rates, and site-specific response dynamics. Azzolin et al.^25^ used a statistical shape model to generate detailed personalized computational models of human atria. Cappon et al.^53^ developed personalized DTs using patient physiology for glucose concentration simulations.

### Disease Applications of Health DTs

We categorized existing studies using the Phecode^65^ category to summarize the applications of DTs across different disease domains as shown in Figure 4-b. The most applications of health DTs are in cardiovascular medicine^16,17,19–25^, such as extracranial carotid artery disease^56^ and aortic aneurysms^18^. Neoplasms are the second largest application domain, with DTs modeling different cancers and associated conditions, such as lung^38^, brain^26–28^, breast^39^, colorectal^66^, metastatic cancers^47^, and cancer-induced pain^60^. Eight studies focused on chronic conditions, such as diabetes (Type 2^40–42,48,52^ and Type 1 diabetes^51,53,54^). Some researchers applied health DTs to study infectious diseases^33,49^, for example, developing the COVID-19 vaccine^45,61^.

About 22% of the studies belong to various other disease domains. In the musculoskeletal domain, DTs were used to improve skull surgery procedures^63^ and fracture management^34,46^. Two studies developed DTs for selecting treatments for respiratory conditions including pneumonia^37^ and neonatal respiratory failure^30^. In gastroenterology, DTs were used for predicting the progression of Crohn’s disease^36^ and portal hypertension^31^. DTs were also applied to predict the onset of brain atrophy^44^ and validate treatments^32^ for multiple sclerosis. Bahrami et al. (2022)^58^ employed a physics-based DT to propose tailored therapy for chronic pain management. In addition to specific disease domains, DTs were also developed for broad health management applications, such as diet and healthy aging^43,50,55,57,64^.

### Data Sources

Data for constructing DTs comes from three primary sources: data from prior clinical studies (58%), real-world data, including electronic health records (EHRs) (22%), and simulation-generated data (20%). Clinical study data include both clinical trial data^30,32,39,51,52,59,60^ and data gathered from observational cohorts^20,36,38,40,42,44,49^. This category covers various types of data, such as structured clinical variables (e.g., conditions and treatments)^31,33,43^, imaging data (e.g., CT scans^57^, specimens^46^, and 3D images^23,34^), and sensory readings^16,17,25^. Among these, only very few datasets (6 datasets used by 5 studies) are publicly accessible (Table 1).

Real-world data are the data relating to patients’ health status collected during routine care, including EHRs and administrative insurance claims data^26,28,37,47,56,61^. Nine real-world datasets (used by six studies) are publicly accessible (Table 1), with the remaining five studies using private data. Some studies use simulated data for testing the DT models across various disease and application domains, including cardiovascular^18,19,21,22^, type 1 diabetes^53^, vertebroplasty procedures^62^, and 3D skull structure^63^. While these simulated data are useful for developing and validating the DT models, they may fall short of creating comprehensive DTs that represent real-world patients and health care settings.

## Discussion

In this review, we elucidated the differences among DTs, MSMs, and ABMs. We then reviewed n=50 existing DT studies in health care and summarized the studies based on the DT types, foundational techniques, applications, and datasets. We identified five types of health DTs, including patient-based DTs (n = 30), organ-based DTs (n = 15), physiological system-based DTs (n=2), procedure-based (n=2), and miscellaneous (n = 1). Most organ-based DTs were designed for forecasting treatment response, while patient-based DTs are primarily used for health monitoring. Regarding foundational techniques of DTs, our review identified 29 studies that employed AI/ML techniques and 18 studies that adopted mathematical models in developing DTs. From the view of applications, most DTs are designed for cardiovascular medicine, Neoplasms, chronic conditions, and infectious diseases. Clinical study data, real-world data, and simulated data have all been used for DT development. Overall, our review shows that AI-based DTs have demonstrated an emerging trend as more and more large-scale datasets, especially large collections of real-world EHR data, have become increasingly available, and computational power has dramatically increased, given the rising capability of Graphics Processing Units (GPUs) and deep neural networks.

Admittedly, there seems to be a clout-chasing phenomenon in a few of the existing studies because of the hot trend of DTs. Some research^22,53,58^ focused more on developing modeling approaches using simulated data rather than proposing a true DT, i.e., linking the virtual model to real-world entities. Although the authors claimed that these methods have the potential to develop health DTs using real-world data, they neglected to explain to what extent their studies could be associated with the DT development process. For instance, a study merely developed models for metastatic disease detection based on radiology reports of three separate organs but did not explain how their models can contribute to creating a DT.^47^ Being able to predict outcomes (or any future events or changes in the system) is a basic need of DTs, but these alone should not be called DTs. Furthermore, some authors may have inaccurately described their studies as creating DTs, when they should have been classified under other simulation techniques. For example, Lin et al. (2023)^66^ claimed to have created patient DTs of 5,417,699 Taiwanese individuals to simulate the effectiveness of colorectal cancer screening as an intervention, rather than conducting true randomized controlled trials. This study seems to be more aligned with the definition of microsimulation models; indeed the authors cited microsimulation as DT, stating, “*the concept of the digital twin was realized in the realm of cancer prevention and screening by the parallel universe approach, which has already been used in a micro-simulation scenario for the development of CISNET (Cancer Intervention and Surveillance Modeling Network)*”^66^. However, it remains unclear whether those virtual patients do correspond to actual patients in the real world or were just generated using simulation parameters derived from the real-world population, leading to the misclassification of their CISNET microsimulation models as “digital twins”.

In addition to the misuse of the terminology, several other issues that need to be carefully considered in creating health DTs, are not adequately addressed in prior research. First, model fairness and bias have not been considered in existing health DT studies^67,68^. Health DTs, corresponding individuals in the real world, should serve everyone fairly, regardless of their socioeconomic status, and should not exacerbate existing health disparities and inequalities. DT developers should assess the potential biases of data used to train the models, as well as the bias that is introduced by the modeling approaches. Bias mitigation would be conducted for identified bias before the health DT application^67^. Furthermore, health DTs rely heavily on the quality and completeness of real-world data from real-world individuals for both model creation and linkage to real-world entities. Lin et al suggested data completeness can generally improve model effectiveness, emphasizing the importance of data quality^69,70^. Another important gap in existing DT research is explainability and transparency; stakeholders often lack insight into how the DT works, leading to concerns about the trustworthiness of the models. Integrating tools that facilitate technical scrutiny of an algorithm’s behavior and its uncertainties is essential and should become a standard practice in algorithm development^71^. Researchers and developers also need to make the DT workflow and models transparent, creating “*white-box*” rather than “*black-box*” systems. Last but not least, it is important to note that currently almost all existing studies are focused on the development of DT modeling approaches. There are still no real-world implementations of health DTs, and no study has been able to make a live connection to continuously achieve the bidirectional data exchange as defined in true DTs, i.e., DTs inform health choices or actions and the individuals’ data feedback to the models with updated information or improvement to the models. To address these research gaps and unmet needs in health DTs, the involvement of all stakeholders, with consideration of human-AI teaming,^72^ is critically needed in the development and co-creation of the health DTs.

Looking ahead, there is a promising future in research focused on developing and implementing health DTs, particularly given the recent rapid evolution in large foundation models, especially in large language models (LLMs) such as ChatGPT and GPT-4. These foundation models have impressive abilities to adapt to various downstream tasks, a desired goal of DTs. Discussions of foundation models for DTs have started in the general domain^73,74^; nevertheless, more research and development work is very much needed, especially for health DTs^74^. From the application standpoint, real-world applications of utilizing DTs, especially for ongoing care and monitoring of chronic diseases, are greatly needed. Other novel uses of health DTs should also be a focus of future research. Furthermore, more and more studies are utilizing deep learning techniques to build up health DT systems. As deep learning models often lack interpretability, a possible solution would be integrating deep learning techniques with prior domain knowledge to improve transparency, in addition to the line of explainability research^75^. Besides, most current studies have built DTs based on data from a single source. The next-generation DTs should incorporate multimodal data (e.g., structured fields from medical records, free-text reports and physician notes, imaging, genomics, and environmental factors) to comprehensively model an entity. The rising interest and ability of multimodal foundation models that can leverage data from multiple modalities might be a critical advancement for building health DTs. Finally, as mentioned above, addressing bias and fairness remains a primary concern, with researchers urged to assess and mitigate potential biases of current health DT techniques.

Our study is subject to several limitations. First, we excluded non-English studies and reports. Second, given that the current scope review is focused on DTs, we reviewed solely review-type articles discussing MSM and ABM, without diving into the individual studies and details of those techniques. Future investigations should comprehensively explore DTs and relevant techniques within specific application domains.

In conclusion, this scoping review offers valuable reference information and perspectives for researchers who are interested in DT techniques and applications in health care, while also highlighting the gaps and future research directions in this field.

## Figures and Tables

**Table 1.** Publicly accessible datasets for health digital twin research.

Data Source	Name	Description	Link
**Clinical Studies**	Human Connectome Project (HCP)	A public research data includes a series of studies that focus on the connections within the human brain.	https://www.humanconnectome.org/
	Alzheimer’s Disease Neuroimaging Initiative (ADNI)	A longitudinal multicenter study designed to develop clinical, imaging, genetic, and biochemical biomarkers for the early detection and tracking of Alzheimer’s disease (AD).	https://adni.loni.usc.edu/
	OhioT1DM dataset	A dataset to facilitate research in blood glucose level prediction. It contains 12 individuals with type 1 diabetes.	http://smarthealth.cs.ohio.edu/OhioT1DM-dataset.html
	Center for Advanced Studies in Adaptive Systems (CASAS) dataset	This dataset consists of real-world sensor data collected from smart home environments.	https://casas.wsu.edu/
	Single heart failure patient with atrial fibrillation	This dataset includes detailed clinical data from a 78-year-old female heart failure patient with atrial fibrillation.	https://zenodo.org/records/7405335
	Human Hepatic Glucose Metabolism	This dataset comprises experimental data on glycogenolysis and glycogen synthesis extracted from various studies.	https://doi.org/10.1371/iournal.pcbi.1002577.s001
**Real-world Data**	Medical Information Mart for Intensive Care -III (MIMIC-III)	A large database contains health-related data associated with over forty thousand patients who stayed in ICU.	https://physionet.org/content/mimiciii/1.4/
	elCU Collaborative Research Database	A large multi-center critical care database contains health data from ICU patients.	https://eicu-crd.mit.edu/
	Whole Brain Atlas	This dataset provides anatomical and functional imaging data of the human brain.	https://www.med.harvard.edu/aanlib/
	Global Initiative on Sharing All Influenza Data (GISAID)	This dataset contains genomic sequences and related clinical and epidemiological data for various influenza viruses and coronaviruses.	https://gisaid.org/
	National Child Development Study (NCDS)	This dataset contains longitudinal data on the lives of individuals born in a single week in 1958 in Great Britain, encompassing a wide range of information.	https://ncds.info/
	Clinical Practice Research Datalink (CPRD)	CPRD contains real-time UK population health data, for epidemiological and pharmacoepidemiological research.	https://www.cprd.com/data
	Cerner Real-World Data (CRWD)	The dataset is a de-identified big data source of multicenter electronic health records.	https://www.oracle.com/health/population-health/real-world-data/
	National Health and Nutrition Examination Survey (NHANES)	This dataset provides comprehensive health and nutritional data from a nationally representative sample of the U.S. population	https://www.cdc.gov/nchs/nhanes/index.htm
	Brain MRI Image dataset from Kaggle	It contains brain MRI Images for brain tumor detection, collected from Google Images.	https://www.kaggle.com/datasets/navoneel/brain-mri-images-for-brain-tumor-detection

## Data Availability

No new or unpublished data is included within the study.
